# Analytical, Numerical and Experimental Study of a Horizontal Electrothermal MEMS Microgripper for the Deformability Characterisation of Human Red Blood Cells

**DOI:** 10.3390/mi9030108

**Published:** 2018-03-02

**Authors:** Marija Cauchi, Ivan Grech, Bertram Mallia, Pierluigi Mollicone, Nicholas Sammut

**Affiliations:** 1 Department of Mechanical Engineering, Faculty of Engineering, University of Malta, MSD 2080 Msida, Malta; pierluigi.mollicone@um.edu.mt; 2 Department of Microelectronics and Nanoelectronics, Faculty of Information and Communications Technology, University of Malta, MSD 2080 Msida, Malta; ivan.grech@um.edu.mt (I.G.); nicholas.sammut@um.edu.mt (N.S.); 3 Department of Metallurgy and Materials Engineering, Faculty of Engineering, University of Malta, MSD 2080 Msida, Malta; bertram.mallia@um.edu.mt

**Keywords:** MEMS microgripper, red blood cells, electrothermal actuation, analytical model, finite element model, fabrication, PolyMUMPs™, temperature, displacement, stresses

## Abstract

Microgrippers are typical microelectromechanical systems (MEMS) that are widely used for micromanipulation and microassembly in both biological and micromanufacturing fields. This paper presents the design, modelling, fabrication and experimental testing of an electrothermal microgripper based on a ‘hot and cold arm’ actuator design that is suitable for the deformability characterisation of human red blood cells (RBCs). The analysis of the mechanical properties of human RBCs is of great interest in the field of medicine as pathological alterations in the deformability characteristics of RBCs have been linked to a number of diseases. The study of the microgripper’s steady-state performance is initially carried out by the development of a lumped analytical model, followed by a numerical model established in CoventorWare^®^ (Coventor, Inc., Cary, NC, USA) using multiphysics finite element analysis. Both analytical and numerical models are based on an electothermomechanical analysis, and take into account the internal heat generation due to the applied potential, as well as conduction heat losses through both the anchor pads and the air gap to the substrate. The models are used to investigate key factors of the actuator’s performance including temperature distribution, deflection and stresses based on an elastic analysis of structures. Results show that analytical and numerical values for temperature and deflection are in good agreement. The analytical and computational models are then validated experimentally using a polysilicon microgripper fabricated by the standard surface micromachining process, PolyMUMPs™ (Durham, NC, USA). The microgripper’s actuation is characterised at atmospheric pressure by optical microscopy studies. Experimental results for the deflection of the microgripper arm tips are found to be in good agreement with the analytical and numerical results, with process-induced variations and the non-linear temperature dependence of the material properties accounting for the slight discrepancies observed. The microgripper is shown to actuate to a maximum opening displacement of 9 μm at an applied voltage of 3 V, thus being in line with the design requirement of an approximate opening of 8 μm for securing and characterising a RBC.

## 1. Introduction

Over the past years, microelectromechanical systems (MEMS) have played a key role in various emerging technologies within the scientific and engineering domains. Microgrippers are typical MEMS devices whose compact size, low power consumption and low cost make them ideal tools in microassembly and micromanipulation fields. The primary function of microgrippers is to handle and manipulate micro-objects, such as micromechanical parts [1,2] and biological cells [3], without causing damage. MEMS microgrippers have been the subject of numerous works in literature, focusing mainly on the microgripper’s design and mechanical structure [4], operational aspects [5], material selection [6], microfabrication [7,8,9,10], and applicability to diverse environments [11].

An essential component of all active MEMS devices is a microactuator that ensures the necessary motions to make the device operational. Different actuation methods include, among others, the use of shape-memory alloys, electrostatic, electrothermal, piezoelectric, pneumatic, and electromagnetic mechanisms [12]. Thermally activated beam flexure has been one of the first developed working principles within the MEMS domain [13,14], mostly owing to the merits of a simple structure that can be made using proven technology with a limited number of fabrication steps. The operating principle of electrothermal actuators relies on the differential thermal expansion of well-designed structures induced via resistive heating. Moreover, electrothermal actuators have several advantages over other actuation mechanisms in that they can generate a relatively large output force and displacement with a small actuation voltage [15].

**Figure 1:**
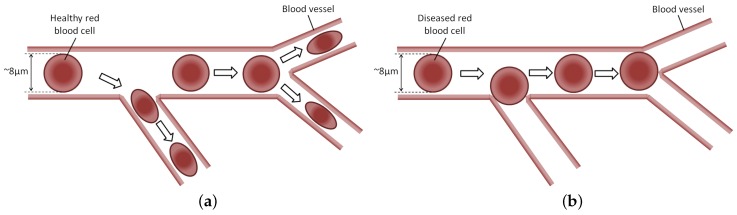
Schematic illustration of red blood cells (RBCs) flowing within a blood vessel. Healthy RBCs can freely flow through the smaller blood vessels due to their ability to undergo cellular deformation (**a**). This is not the case for diseased RBCs whose impaired deformability characteristics do not allow them to pass through the smaller blood vessels (**b**).

MEMS microgrippers play an important role in the manipulation and characterisation of cells and tissues for biomedical applications as demonstrated by these studies [16,17,18,19]. This paper presents an electrothermally actuated microgripper suitable for the handling and deformability characterisation of human red blood cells (RBCs). The primary function of RBCs is to carry oxygen from the lungs to the body tissues, as well as carbon dioxide (CO${}_{2}$), as a waste product, away from the tissues and back to the lungs. Under physiological (i.e., healthy) conditions, RBCs possess a unique capacity to undergo cellular deformation to navigate across various human microcirculation vessels. Such deformability characteristics of RBCs allow the flow of a single, biconcave disk-shaped RBC, with approximate dimensions of 8 μm in diameter and 2 μm in thickness, through capillaries with smaller diameters, in the process supplying oxygen to tissues of the body (Figure 1). Impaired RBC deformability due to pathological factors including among others malaria, sickle cell anemia, diabetes, hereditary disorders, paroxysmal nocturnal hemoglobinuria, and myocardial infarction (commonly known as a heart attack), hinders their function of oxygen delivery to biological tissues due to the increased cell membrane stiffness, and can thus be an important indicator of human well-being [20]. In the case of healthy cells, the gripping force lies in the level of a few microNewtons [15]. The grasping force is expected to increase to achieve the same cell deformation in diseased cells due to the increased membrane stiffness. Due to its pathophysiological importance, measurement of the deformability of RBCs has been the focus of numerous studies over the past decades [21]. Moreover, different laboratory procedures have been developed to enable RBCs to be reliably tested in air [22].

This paper presents the analytical and numerical modelling, fabrication and testing of an electrothermal MEMS microgripper designed for the characterisation of RBCs. Section 2 briefly introduces the microgripper design and its operating principle, while Section 3 describes the surface micromachining process used to fabricate the microgripper in this work. The analytical model is developed in Section 4 where the electrothermal performance of the actuator is studied, followed by a thermomechanical analysis to describe the microgripper’s mechanical performance. Section 5 presents the developed numerical model of the actuator together with the finite element analysis (FEA) modelling techniques implemented within it. An overview of the experimental setup used for the optical microscopy studies of the fabricated microgripper is given in Section 6. The analytical, numerical and experimental results for the thermal and structural analyses of the microgripper are then presented and compared in Section 7. Finally, Section 8 summarises the findings of this study, presents the concluding remarks, and addresses future research directions.

## 2. Microgripper Design and Operating Principle

**Figure 2:**
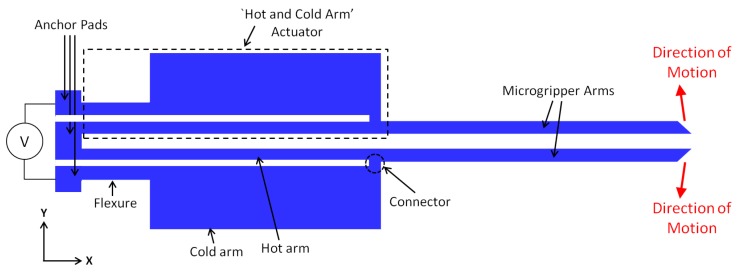
Schematic diagram of the microgripper design (not-to-scale) consisting of two ‘hot and cold arm’ actuators that are placed anti-symmetrically next to each other and that are electrically connected in an in series configuration.

The microgripper presented in this work is based on the ‘hot and cold arm’ actuator design and the electrothermal actuation principle. This actuator consists of two parallel arms of different widths that are joined at their free ends to form a U-shape electrical loop. A complete microgripper consists of two such actuators placed anti-symmetrically next to each other, with two extending microgripper arms at their ends to amplify the achieved displacement (Figure 2).

Electrothermal actuation relies on the thermal expansion of the hot and cold arms that is achieved through resistive heating of the suspended polysilicon structure. With the flow of an electric current, the narrower arm experiences greater resistive heating and thermal expansion than the wider arm, resulting in a bending moment and in lateral displacement of the actuator tip in the direction of the cold arm. The actuator movement is influenced by the temperature difference between the narrower (and hotter) arm segment and the wider (and colder) arm segment. Increasing this temperature difference improves the operating efficiency of the horizontal electrothermal actuator in terms of a larger in-plane displacement achieved with the same applied voltage. It is generally desired to design a horizontal MEMS microgripper with a large in-plane displacement and a zero (or minimal) out-of-plane deflection.

The main design variables that determine the performance of a microgripper are its geometric parameters (e.g., length, width and thickness) and its material properties (e.g., electrical and thermal conductivities, coefficient of thermal expansion, etc.). In this work, only the lateral dimensions i.e., the length and width of the microgripper could be defined by the user as the mechanical and sacrificial film layer thicknesses were constrained by the fabrication process. Although the material properties such as the thermal and electrical conductivities are dependent on the doping concentration of the microgripper material, the latter was set by the fabrication process, and therefore, the material properties were also considered to be fixed parameters. The microgripper structure is derived from the optimised design presented in [23] to ascertain that the flexure is designed long enough to ensure proper elastic deflection of the actuator. The function of the flexure is to effectively operate as a low-stiffness extension of the otherwise stiff cold arm, allowing the entire actuator to achieve the required deflection. The flexure is also often manufactured with the same width as the hot arm, however its relatively shorter length restrains its temperature from increasing to the same degree as that of the hot arm.

## 3. Fabrication Process

The studied microgripper is designed for fabrication using one of the Multi-User MEMS Processes (MUMPs), known as PolyMUMPs^TM^ [24]. PolyMUMPs^TM^ is a surface micromachining process that provides three structural layers of polysilicon, two sacrificial layers of phosphosilicate glass (PSG), a silicon nitride layer for electrical isolation between the polysilicon and the silicon substrate, and a metal layer for bonding, probing or electrical routing. The polysilicon and PSG layers are deposited by low pressure chemical vapour deposition, and are subsequently lithographically patterned and etched by reactive ion etching to obtain the final wafer pattern. The metal layer consists of a thin adhesion layer of chromium and 0.5 μm of gold, and is deposited onto the surface of the polysilicon beam at the end of the process and pattered using lift-off. The metal layer can be selectively deposited on the structure to enhance thermal expansion. However, this might also result in an increase in the beam’s out-of-plane displacement due to the creation of a bimorph structure [25]. The structure is finally released by immersing the chip in a standard hydrofluoric (HF) solution designed to fully remove the sacrificial PSG layers from the fabricated structure. This is followed by supercritical CO${}_{2}$ drying of the released structure to reduce stiction.

**Figure 3:**
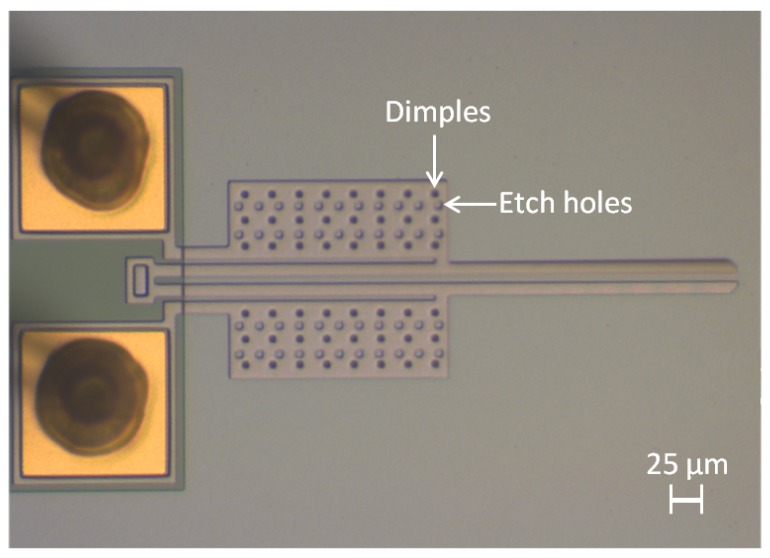
Optical microscope image of the fabricated polysilicon microgripper, also showing the gold wire bond interconnections on the probe pads for electrical activation of the structure.

**Figure 4:**
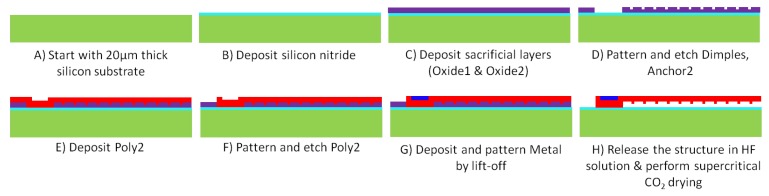
Schematic cross-sectional view (not-to-scale) of the PolyMUMPs™ process flow for fabrication of the microgripper structure. This is based on the microfabrication process described in [24]. The air gap resulting from stacked Oxide1 and Oxide2 layers is 2.75 μm.

The microgripper fabricated in this study consists of a PolyMUMPs™ structure made out of one polysilicon structural layer (Poly2) with anchored metal probe pads at one end of the actuators. The microgripper was mounted on an integrated circuit (IC) package, and ultrasonic wire bonding was utilised to provide electrical connections through gold wires between the probe pads on the microgripper and the contact pads on the package. The fabricated microgripper is shown in Figure 3, its geometrical dimensions are given in Table 1 and the fabrication process steps are illustrated in Figure 4.

## 4. Analytical Model

### 4.1. One-Dimensional Model

**Figure 5:**
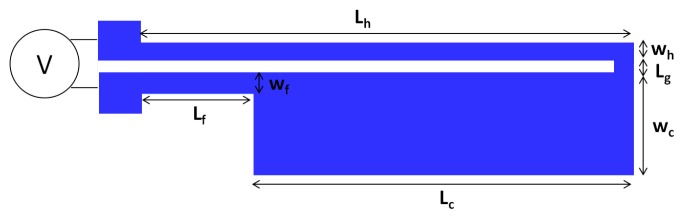
Top view of the electrothermal actuator with labelled dimensions.

**Figure 6:**
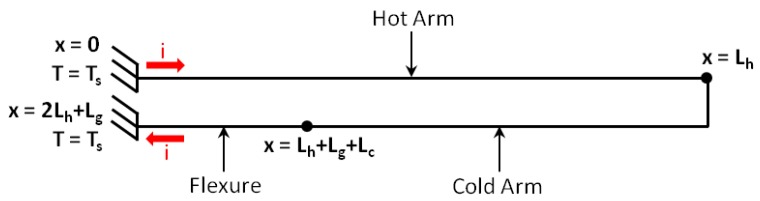
Simplified 1D model of the actuator, showing the direction of the current, *i*. The anchor pads are represented by fixed supports that are assumed to be at the same temperature of the substrate, $T_{s}$.

The reliable prediction of the thermal actuator’s displacement primarily requires the accurate modelling of the temperature distribution within the device. An inherent characteristic of a surface micromachining process is that the size of the fabricated device’s cross-section is generally much smaller than its length. The temperature variation within the arms’ cross-section can in fact be considered negligible with respect to the temperature variation along the length of each arm. In this regard, the electrothermal analysis of the ‘hot and cold arm’ actuator can be simplified as a one-dimensional (1D) problem to model the longitudinal heat flow along the polysilicon beam [26]. It should also be noted that since the length of the connector between the hot arm and the cold arm, $L_{g}$, is much smaller than the individual lengths of the hot arm ($L_{h}$), cold arm ($L_{c}$) or flexure ($L_{f}$), it can either be assumed to be zero or added to the length of the cold arm in the analytical model. In this way, each actuator arm can be modelled as three line-shape microbeams (hot arm, cold arm and flexure) connected in series (Figure 5 and Figure 6).

### 4.2. Electrothermal Analysis

The 20 μm thick silicon substrate is considered as a huge thermal mass at room temperature, $T_{s}$. The actuator’s probe pads which are anchored to the substrate thus act as heat sinks, and are fixed at room temperature in the model. Moreover, the small dimensions of the actuator allow the bottom surface’s convective heat transfer coefficient to be well approximated by conduction, through the stagnant air layer beneath it, to the substrate. Due to the very small gap between the substrate and the polysilicon beam ($t_{a}$ = 2.75 μm), conduction from the beam’s bottom surface, through the air and nitride layers, to the substrate dominates in relation to other heat losses by convection and radiation. In fact, heat losses by convection from the other beam surfaces can reasonably be ignored for this thermal actuator operated in still air [27]. Additionally, the amount of thermal radiation emitted by a structure is given by σ ${AT}^{4}$, where σ is the Stefan-Boltzmann constant, *A* is the surface area, and *T* is the temperature. Since this actuator is not designed for routine operation at high operational power (thus temperatures are relatively low), and since the area of the actuator is extremely small, the influence of thermal radiation on the deflection of the thermal actuator is also not significant and thus can reasonably be assumed negligible in this work [25]. The principal sources of heat losses for thermal actuators operated close to room temperature in still air are thus conduction through the polysilicon beam’s anchor pads, and through the air gap and nitride layer, to the substrate.

**Figure 7:**
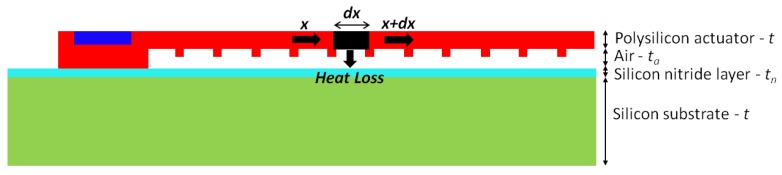
Cross-section of the actuator for the 1D thermal analysis (not-to-scale), illustrating the differential element used to derive the heat flow equation.

The heat flow equation is derived by examining a differential element of the microbeam of thickness *t*, width *w* and length dx (Figure 7) [28]. The transient response time of the actuator is very small such that the steady-state condition is reached almost immediately after the voltage is applied. When the heat transfer flow is under steady-state, the internal heat generation in the element due to the applied potential is dissipated by means of heat conduction out of the element as follows:

(1) $$-k_{p}wt{\left[\frac{dT}{dx}\right]}_{x}+J^{2}\rho wtdx=-k_{p}wt{\left[\frac{dT}{dx}\right]}_{x+dx}+\frac{Sw(T-T_{s})dx}{R_{T}}$$

where *w* and *t* are the width and thickness of the element in the polysilicon layer respectively, *T* and $T_{s}$ are the beam and substrate temperatures respectively, $k_{p}$ is the thermal conductivity of polysilicon, *J* is the current density, ρ is the resistivity of polysilicon, and *S* is the shape factor which accounts for the impact of the shape element on heat conduction to the substrate. This geometric factor represents the ratio of the heat loss from the sides and bottom of the beam, to the heat loss from the bottom of the beam only [26], and thus accounts for conduction from the sides of the beam through the surrounding air to the substrate. *S* is given by:

(2) $$S=\frac{t}{w}\left(2\frac{t_{a}}{t}+1\right)+1$$

where *w* and *t* are as described previously, and $t_{a}$ is the thickness of the air gap between the polysilicon beam and the nitride layer.

The thermal resistance between the polysilicon beam and the substrate is given by $R_{T}$ as follows:

(3) $$R_{T}=\frac{t_{a}}{k_{a}}+\frac{t_{n}}{k_{n}}$$

where $t_{a}$ is as described previously, $t_{n}$ is the thickness of the nitride layer, and $k_{a}$ and $k_{n}$ are the thermal conductivity of air and the nitride layer respectively.

The current density *J* is expressed as follows:

(4) $$J=\frac{V}{\rho L}$$

where *V* is the voltage applied across the actuator arm, ρ is as described previously, and *L* is the length of polysilicon beam that the current passes through.

After taking the limit as dx→ 0, Equation (1) gives the following second order differential equation:

(5) $$k_{p}\frac{d^{2}T}{dx^{2}}+J^{2}\rho =\frac{S(T-T_{s})}{R_{T}t}$$

The first and second terms on the left hand side of Equation (5) represent the net rate of heat conduction into the element per unit volume, and the rate of heat energy generation inside the element per unit volume, respectively. The right hand side of the thermal model equation represents the rate of heat energy loss by conduction in the element per unit volume.

Equation (5) can be simplified as follows:

(6) $$\frac{d^{2}\theta \left(x\right)}{dx^{2}}-m^{2}\theta \left(x\right)=0$$

with:

(7a) $$\theta \left(x\right)=T\left(x\right)-T_{\theta }$$

(7b) $$T_{\theta }=T_{s}+\frac{J^{2}\rho }{k_{p}m^{2}}$$

(7c) $$m^{2}=\frac{S}{k_{p}tR_{t}}$$

where $T_{H}$ and $m_{h}$ are equal to $T_{\theta }$ and *m* with *w* = $w_{h}$, respectively; similarly $T_{C}$ and $m_{c}$ are equal to $T_{\theta }$ and *m* with *w* = $w_{c}$, respectively; $w_{h}$ and $w_{c}$ are the widths of the hot arm and the cold arm respectively, and $T_{F}$ = $T_{H}$ and $m_{f}$ = $m_{h}$ since the width of the flexure is equal to the width of the hot arm for this actuator design.

Equation (6) is a linear differential equation with the following general solutions for the temperature distribution of the hot arm $T_{h}$, cold arm $T_{c}$, and flexure $T_{f}$:

(8) $$T_{h}\left(x\right)=T_{H}+C_{1}e^{m_{h}x}+C_{2}e^{-m_{h}x}$$

(9) $$T_{c}\left(x\right)=T_{C}+C_{3}e^{m_{c}x}+C_{4}e^{-m_{c}x}$$

(10) $$T_{f}\left(x\right)=T_{F}+C_{5}e^{m_{f}x}+C_{6}e^{-m_{f}x}$$

The above temperature distributions are to be defined by calculating the constants $C_{1}$ to $C_{6}$ based upon the boundary conditions applied to the microbeam. The boundary conditions are obtained by utilising the continuity of both temperature *T*, and the rate of heat conduction $\dot{q}$ across the joint points of the hot arm, cold arm and flexure. They are given by:

- At $x=0,T_{h}\left(x\right)$ = $T_{s}$
- At $x=L_{h}$, $T_{h}\left(x\right)$ = $T_{c}\left(x\right)$
- At $x=L_{h}$+$L_{g}$+$L_{c}$, $T_{c}\left(x\right)$ = $T_{f}\left(x\right)$
- At $x=2L_{h}$+$L_{g}$, $T_{f}\left(x\right)$ =$T_{s}$
- At $x=L_{h}$, ${\dot{q}}_{h}\left(x\right)$ = ${\dot{q}}_{c}\left(x\right)$
- At $x=L_{h}$+$L_{g}$+$L_{c}$, ${\dot{q}}_{c}\left(x\right)$ = ${\dot{q}}_{f}\left(x\right)$

The results of this electrothermal analysis are used as an input for the thermomechanical analysis described in Section 4.3. The thermomechanical analysis uses the average temperatures of the hot arm, cold arm and flexure to compute the thermal strains of the respective arms, and then employs the method of virtual work to predict the actuator deflection. By integrating Equations (8)–(10) and dividing each by the respective beam length, the average temperatures of the hot arm $\bar{T_{h}}$, cold arm $\bar{T_{c}}$, and flexure $\bar{T_{f}}$, can be expressed as follows:

(11) $$\bar{T_{h}}=T_{H}+\frac{C_{1}}{m_{h}L_{h}}\left(e^{m_{h}L_{h}}-1\right)-\frac{C_{2}}{m_{h}L_{h}}\left(e^{-m_{h}L_{h}}-1\right)$$

(12) $$\bar{T_{c}}=T_{C}+\frac{C_{3}}{m_{c}\left(L_{c}+L_{g}\right)}\left(e^{m_{c}\left(L_{h}+L_{g}+L_{c}\right)}-e^{m_{c}L_{h}}\right)-\frac{C_{4}}{m_{c}\left(L_{c}+L_{g}\right)}\left(e^{-m_{c}\left(L_{h}+L_{g}+L_{c}\right)}-e^{-m_{c}L_{h}}\right)$$

(13) $$\bar{T_{f}}=T_{H}+\frac{C_{5}}{m_{h}L_{f}}\left(e^{m_{h}\left(2L_{h}+L_{g}\right)}-e^{m_{h}\left(L_{h}+L_{g}+L_{c}\right)}\right)-\frac{C_{6}}{m_{h}L_{f}}\left(e^{-m_{h}\left(2L_{h}+L_{g}\right)}-e^{-m_{h}\left(L_{h}+L_{g}+L_{c}\right)}\right)$$

### 4.3. Thermomechanical Analysis

**Figure 8:**
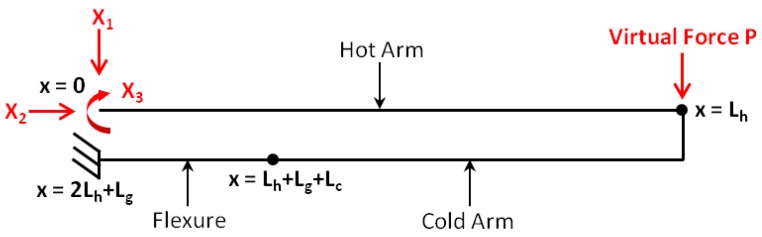
The simplified plane rigid frame for the thermal actuator structure showing the three redundants $X_{1}$, $X_{2}$ and $X_{3}$. The virtual force *P* applied at the free end of the actuator as part of the force method is also indicated.

The structure of the thermal actuator can be considered as a plane rigid frame with two fixed supports (Figure 6). It can be observed that the actuator structure is statically indeterminate with a degree of indeterminacy of 3. The chosen redundant forces/moments are $X_{1}$, $X_{2}$ and $X_{3}$ as shown in Figure 8. The force method [29] is applied to analyse the bending moment of the actuator due to these 3 redundant loads, and $X_{1}$, $X_{2}$ and $X_{3}$ are obtained by solving the following set of simultaneous equations:

(14) $$\left[\begin{array}{ccc} f_{11} & f_{12} & f_{13}\\ f_{21} & f_{22} & f_{23}\\ f_{31} & f_{32} & f_{33} \end{array}\right]\left[\begin{array}{c} X_{1}\\ X_{2}\\ X_{3} \end{array}\right]=\left[\begin{array}{c} 0\\ \Delta L_{h}-\Delta L_{c}-\Delta L_{f}\\ 0 \end{array}\right]$$

where the terms $f_{ij}$ represent flexibility coefficients that define the deflection at *i* due to a unit load at *j*. According to Maxwell’s Law of Reciprocal Deformations, the deflection at *i* due to a unit load at *j* is equal to the deflection at *j* due to a unit load at *i* (as long as the structure is linear elastic). Thus, $f_{ij}$ = $f_{ji}$ and the flexibility matrix in Equation (14) is symmetric. The flexibility coefficients are determined using Equation (15) where $m_{i}$ and $m_{j}$ represent the bending moments due to the respective three unit redundants $X_{1}$, $X_{2}$ and $X_{3}$:

(15) $$f_{ij}=\int_{L}\frac{m_{i}m_{j}}{EI}dx$$

The flexibility coefficients are given for the actuator investigated in this paper by Equations (16a)–(16f):

(16a) $$f_{11}=\frac{1}{3EI_{h}}\left({L_{h}}^{3}+{L_{f}}^{3}\right)+\frac{1}{3EI_{c}}\left(3{L_{h}}^{2}L_{g}+{L_{h}}^{3}-{L_{f}}^{3}\right)$$

(16b) $$f_{12}=f_{21}=-\frac{1}{2EI_{h}}\left({L_{f}}^{2}L_{g}\right)-\frac{1}{2EI_{c}}\left(L_{h}{L_{g}}^{2}+{L_{h}}^{2}L_{g}-{L_{f}}^{2}L_{g}\right)$$

(16c) $$f_{13}=f_{31}=-\frac{1}{2EI_{h}}\left({L_{h}}^{2}+{L_{f}}^{2}\right)-\frac{1}{2EI_{c}}\left({L_{h}}^{2}+2L_{h}L_{g}-{L_{f}}^{2}\right)$$

(16d) $$f_{22}=\frac{1}{3EI_{c}}\left({L_{g}}^{3}+3L_{c}{L_{g}}^{2}\right)+\frac{1}{EI_{h}}\left(L_{f}{L_{g}}^{2}\right)$$

(16e) $$f_{23}=f_{32}=\frac{1}{2EI_{c}}\left({L_{g}}^{2}+2L_{g}L_{c}\right)+\frac{1}{EI_{h}}\left(L_{g}L_{f}\right)$$

(16f) $$f_{33}=\frac{1}{EI_{h}}\left(L_{g}+L_{f}\right)+\frac{1}{EI_{c}}\left(L_{c}+L_{g}\right)$$

where *E* is the Young’s modulus of polysilicon, $I_{h}$ is the moment of inertia for the hot arm and the flexure (since they have the same cross-sectional area), and $I_{c}$ is the moment of inertia for the cold arm.

The total change in length for each of the hot arm $\Delta L_{h}$, cold arm $\Delta L_{c}$, and flexure $\Delta L_{f}$, is composed of the thermal expansion component, and the mechanical component resulting from the expansion restriction. Given the assumed direction of the axial force $X_{2}$ on the three elements (Figure 8), the total respective length changes are given by:

(17) $$\Delta L_{h}=\alpha L_{h}\left(\bar{T_{h}}-T_{s}\right)-\frac{X_{2}L_{h}}{Etw_{h}}$$

(18) $$\Delta L_{c}=\alpha L_{c}\left(\bar{T_{c}}-T_{s}\right)+\frac{X_{2}L_{c}}{Etw_{c}}$$

(19) $$\Delta L_{f}=\alpha L_{f}\left(\bar{T_{f}}-T_{s}\right)+\frac{X_{2}L_{f}}{Etw_{h}}$$

Once the three redundants $X_{1}$, $X_{2}$ and $X_{3}$ are obtained by solving Equation (14), the bending moment of the hot arm due to the thermal expansion can be represented by:

(20) $$M=X_{1}x-X_{3}$$

The deflection of the actuator tip is then obtained by applying a virtual unit force *P* to the free end of the actuator resulting in the following bending moment of the hot arm:

(21) $$\bar{M}=P\left(L_{h}-x\right)$$

Based on the method of virtual work [29], the deflection at the free end of the actuator is given by:

(22) $$u=\int_{0}^{L_{h}}\frac{\bar{M}M}{EI_{h}}=\frac{{L_{h}}^{2}}{6EI_{h}}\left(X_{1}L_{h}-3X_{3}\right)$$

In the actual microgripper design, this displacement is amplified by the extending microgripper arms such that the displacement at the microgripper arm tip, $u_{tip}$, is determined by extrapolating the deflection at the free end of the actuator, *u*, over the length of the microgripper arm, $L_{a}$, as given below:

(23) $$u_{tip}=\frac{{L_{h}}^{2}}{6EI_{h}}\left(X_{1}L_{h}-3X_{3}\right)+\frac{L_{a}L_{h}}{2EI_{h}}\left(L_{h}X_{1}-2X_{3}\right)$$

## 5. Numerical Model

**Figure 9:**
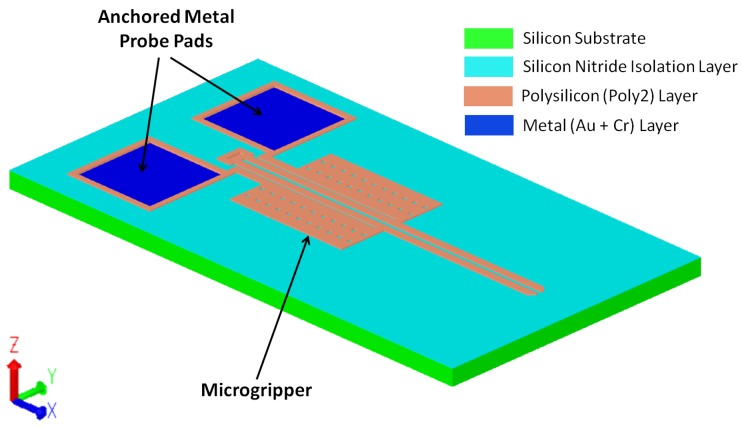
A 3D model of the electrothermal microgripper in Coventorware^®^.

A finite element model that numerically computes and solves the combined electrical, thermal and mechanical behaviour of the designed microgripper is developed using Coventorware^®^. A three-dimensional (3D) model of the designed microgripper is shown in Figure 9.

Similarly to the analytical model, the numerical model is divided into two sections which can be solved sequentially: the electrothermal and thermomechanical analyses. The electrothermal analysis consists of converting the potential distribution values calculated by the solver to data points that reflect the Joule heating effects resulting from the current. The solver determines the temperature distribution and applies the computed temperature values along the actuator beams. The thermomechanical analysis then uses the increase in beam temperatures to compute the thermal strain and the resulting deflection.

The solver uses the material properties specified in the model’s database to compute the electrothermomechanical solution. The material properties implemented within the model are isotropic, and are given in Table 2. Additionally, the numerical model requires boundary conditions to be applied within the electrical, thermal and mechanical domains. In the electrical domain, the actuating voltage was applied across the anchor pads of the actuator arms. The thermal boundary conditions consisted of applying a homogeneous room temperature (300 K) as an initial condition on all model elements, setting the anchor pads to the constant substrate temperature ($T_{s}$ = 300 K) throughout the analysis, and applying a convective heat transfer coefficient to the bottom and side surfaces of the actuator arms to simulate heat losses through the air gap and nitride layer to the substrate. Finally, all anchor pads were fixed in all directions within the mechanical domain.

## 6. Experimental Setup

**Figure 10:**
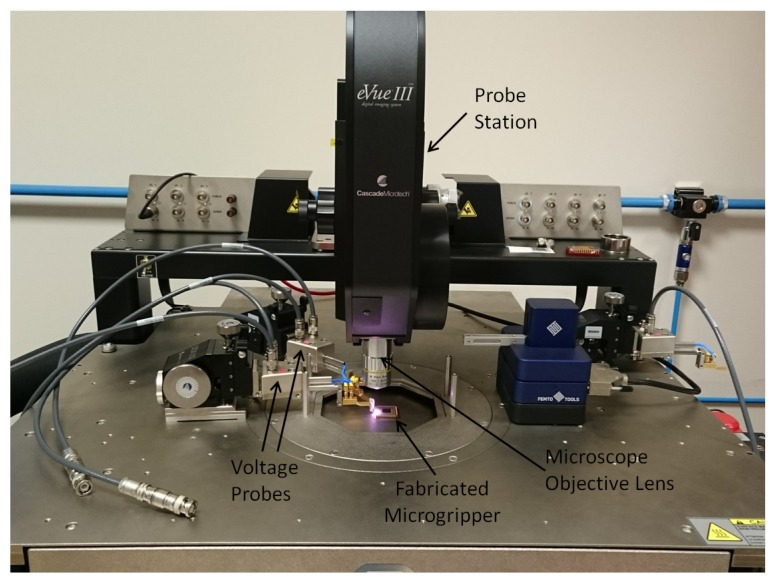
The fabricated microgripper enclosed in an integrated circuit (IC) package and mounted on the platform of the vibration isolation Cascade Microtech probe station with voltage probes to actuate the microgripper structure.

The released microgripper structure manufactured by the fabrication process described in Section 3 was mounted on an IC package to facilitate experimental testing and characterisation. Electrical connections between the power supply and the contact pads of the IC package were established using voltage probes, each having a 3-axis positioning system (Figure 10). Two multimeters were used to measure the voltage and current applied to the structure. The in-plane deflection of the electrothermal microgripper arm tips was measured using an optical microscope-based vision system with a camera embedded within the vibration isolation probe station. Microscope objective lenses with different magnifications were used for the experimental characterisation. Each microgripper arm was actuated by applying an electrical potential difference ranging from 0–3 V in steps of 0.5 V between the hot and cold arm terminals. The interval between each step was more than 5 s to ensure that steady-state conditions were attained. The width of the gripping arm (9 μm) was measured by the optical microscope and was taken as the reference for calibration.

## 7. Results

### 7.1. Thermal Analysis

**Figure 11:**
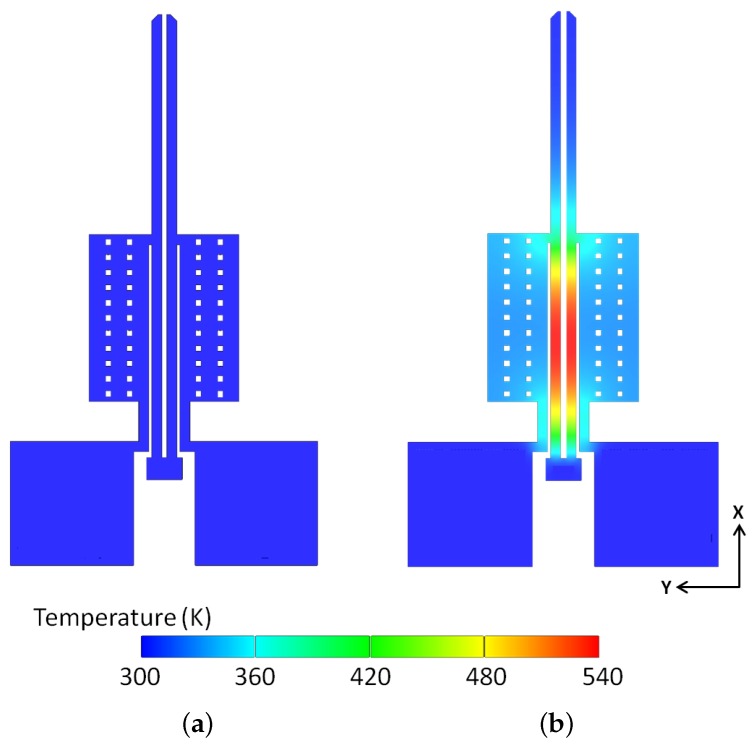
Simulated steady-state temperature profile of the polysilicon electrothermal microgripper: at no applied potential (**a**), and when each arm is subjected to an applied potential of 3 V (**b**). The maximum temperature at the applied potential of 3 V is 540 K and is located near the middle of the hot arms.

An important design constraint imposed on the microgripper’s performance is the maximum temperature reached within the microgripper structure under an applied potential. It must be ensured that the peak temperature of the polysilicon-based electrothermal microgripper stays within the material limits. In this study, the peak temperature is limited to 540 K. This is because higher temperatures might result in issues between the gold and polysilicon layers due to the formation of low-temperature eutectics which will induce the loss of the metallised gold layer. Moreover, in biomedical applications, the maximum operating temperature of the microgripper plays a critical role due to the manipulation of living cells and biological tissues. It can be observed from Figure 11 that the temperature at the microgripper arm tips under an applied potential of 3 V is still near room temperature, thus ensuring the safe handling of any biological species.

**Figure 12:**
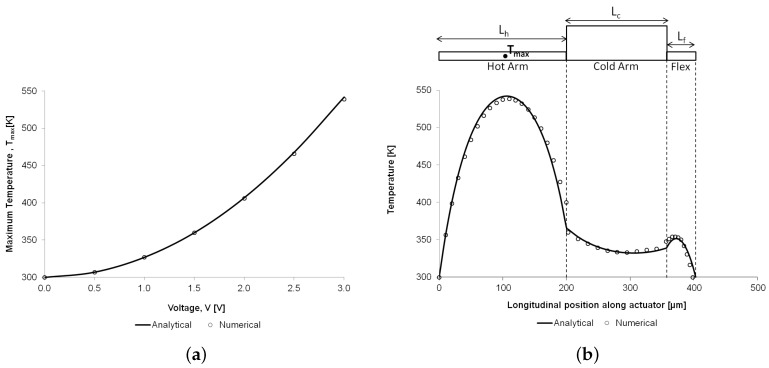
Comparison of the analytical and numerical results for the temperature distribution on the microgripper structure: variation of the maximum temperature on the hot arm as a function of the applied potential (**a**), and temperature profile along the hot arm, cold arm and flexure components at 3 V (**b**). For illustration purposes, the diagram above the graph in (**b**) shows a thermal actuator (without the extending microgripper arms) as if it were unfolded.

Thermal characterisation of the microgripper as a function of the applied voltage was not conducted experimentally in this study due to the absence of the necessary equipment (such as microthermal microscope) to obtain such data. However, the analytical and numerical models developed in this paper were used to predict the peak temperature and to establish if the peak temperature material constraint was violated. Analytical and numerical results for the maximum temperature reached on the hot arm, as well as for the temperature distribution along the hot arm, cold arm and flexure components, are compared and are found to be in close agreement (Figure 12). The analytical temperature distributions along the actuator arms were calculated using Equations (8)–(10) for the hot arm, cold arm and flexure respectively.

### 7.2. Structural Analysis

**Figure 13:**
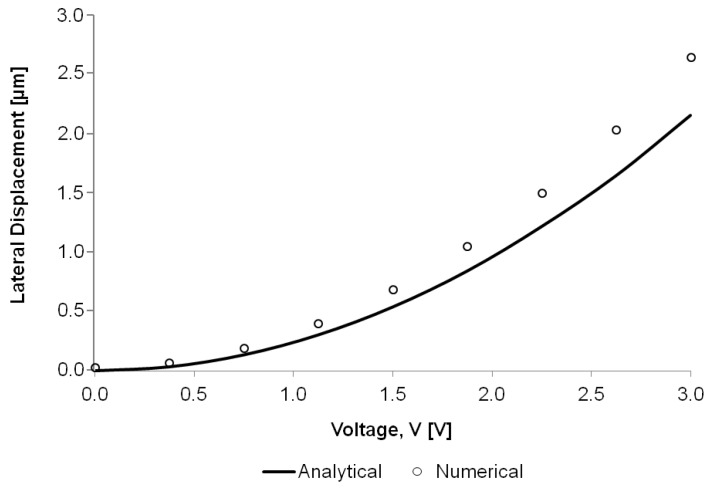
Comparison of the analytical and numerical results for the maximum lateral displacement of the microgripper arm tip as a function of the applied potential.

The different thermal expansions of the hot and cold arms upon applying a potential across the actuator’s probe pads cause the tip of the actuator to move laterally in an arching motion towards the ‘cold’ arm side. This work deals with a horizontal actuator and thus the in-plane displacement is of the utmost interest. The out-of-plane displacement is in fact negligible when compared to the in-plane displacement obtained from this design. The steady-state displacement at the microgripper arm tip is obtained numerically and good agreement is observed with the analytical results calculated using Equation (23) (Figure 13).

**Figure 14:**
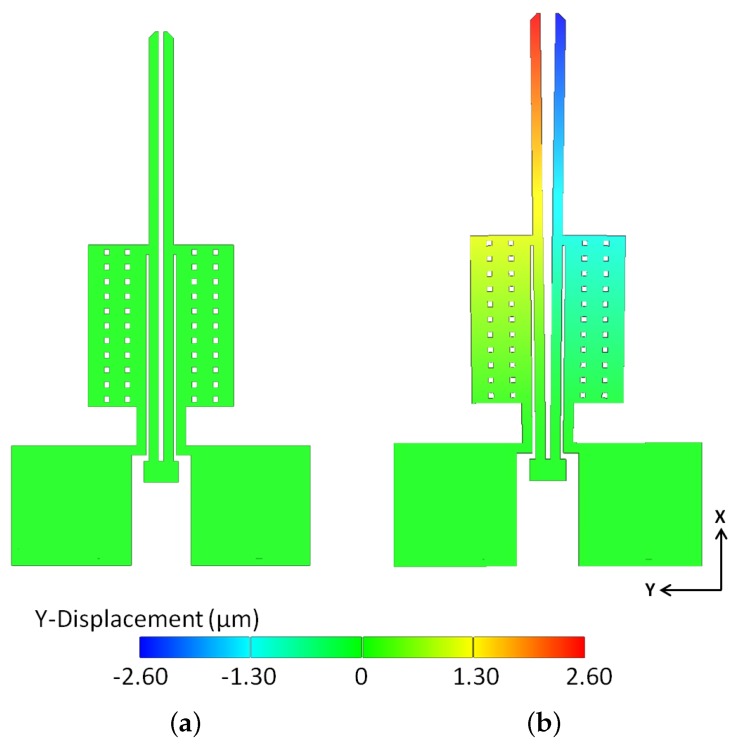
Simulated steady-state lateral displacement profile of the polysilicon electrothermal microgripper: at no applied potential (**a**), and when each arm is subjected to an applied potential of 3 V (**b**). The gap opening in the closed position is 5 μm (**a**). At 3 V, each arm moves laterally by 2.6 μm such that the total gap opening at 3 V is 10.2 μm (**b**).

**Figure 15:**
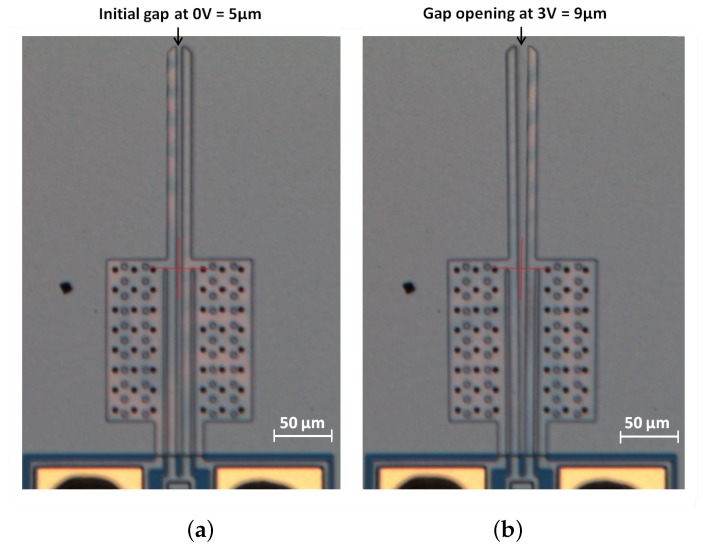
Optical images showing the fabricated polysilicon microgripper when not actuated (**a**), and when each arm is actuated under an applied potential of 3 V (**b**). The gap opening in the closed position is 5 μm (**a**). A potential of 3 V applied across each microgripper arm results in a total gap opening of 9 μm. (**b**)

The analytical and computational models were then validated using experiments performed under atmospheric pressure. It can be observed from Figure 14 andFigure 15 that the predictions of the numerical model match very well with the experimental results. As shown in Figure 15, the actual achievable deflection at 3 V is 9 μm. This is marginally higher than the design specification (8 μm) and therefore, the fabricated microgripper can perform the desired function.

The slight discrepancies observed between the numerical and experimental results for the microgripper arm tip displacement can be due to a number of factors including process-related variations in the electrical, thermal, and mechanical properties of polysilicon, as well as in the resistivity of the material across the wafer. Moreover, the non-linear temperature dependence of properties such as the thermal conductivity, the coefficient of thermal expansion and the convective heat transfer coefficient was not taken into account in the analytical and numerical models. At lower voltages (or current) as is the case in this work, the non-linear material properties that vary with temperature can reasonably be assumed to be constant due to the relatively low temperatures within the microgripper structure. This however can no longer be validly assumed as the peak temperature increases, and capturing this non-linear temperature dependence behaviour becomes highly important when modelling devices at high operating temperatures.

**Figure 16:**
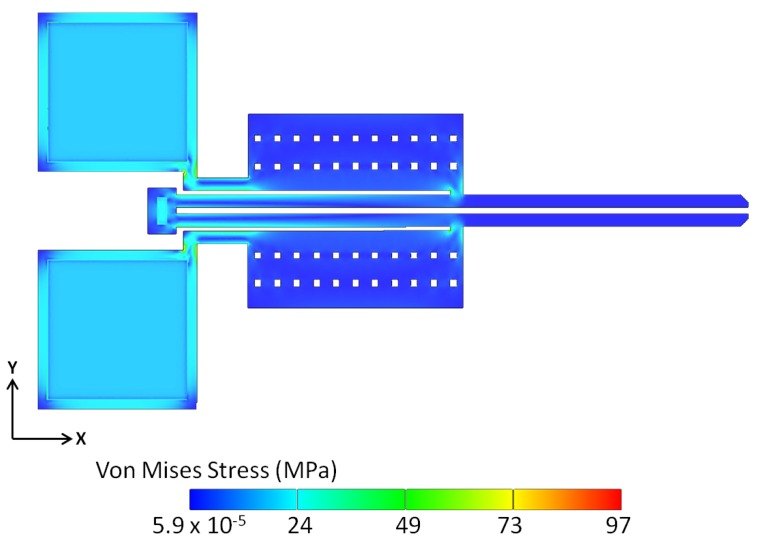
Von Mises stresses developed within the polysilicon microgripper structure at a potential of 3 V.

An important consideration in the analysis of the microgripper’s performance is the investigation of the developed maximum stresses during actuation, in terms of both magnitude and location. As observed in Figure 16, the maximum stresses developed within the microgripper structure at the maximum applied potential of 3 V are mainly located where the hot arms join the anchor pads, and at the connector joining the hot and cold arms. At the potential of 3 V, the maximum Von Mises stress approaches 97 MPa, which is far less than the value of allowable stress. The fracture strength for polysilicon has been found to be 2.9 ± 0.5 GPa in tensile tests and 3.4 ± 0.5 GPa in bending tests [30]. However, the buildup of such stress concentrations can still be mitigated by slight design improvements and geometry modifications including the use of round instead of sharp corners at the highlighted locations.

## 8. Conclusions

The work presented in this paper focuses on the contribution in the analytical and numerical modelling, fabrication, and experimental testing of a horizontal MEMS microgripper designed for the deformability characterisation of RBCs. The developed microgripper is based on a ‘hot and cold arm’ actuator design, utilises electrothermal actuation, and is designed to be compatible with the handling of biological cells by ensuring a very low temperature at the microgripper arm tips during operation. The operating principle of a horizontal electrothermal microgripper is the asymmetrical thermal expansion of a microstructure with variable cross-sections. This differential thermal expansion causes the actuator to move laterally (parallel to the substrate), with the flexure part aiding the flexibility in the direction of motion.

The performance of a polysilicon electrothermal actuator is directly linked to its geometry and process parameters, as well as the input power. This paper has presented the detailed development of an analytical model of the actuator which investigates the relationship between these different factors, together with a numerical 3D model developed using FEA in CoventorWare^®^. The current work establishes these models based on an electrothermomechanical analysis in which heat conduction to the substrate, through the air and nitride layers, and through the anchor pads, is considered the dominant heat loss transfer mechanism. The developed models are intended to predict the behaviour of an electrothermal microgripper at atmospheric pressure and at low operating temperatures, and FEA results are found to be in good agreement with temperature and deflection results obtained from the lumped analytical model. Moreover, the maximum temperature and stresses developed within the microgripper structure are well within the allowable material limits, confirming that the designed microgripper owns enough strength to achieve a stable opening and closing motion.

The microgripper was fabricated with the standard PolyMUMPs^TM^ microfabrication process using a 1.5 μm thick polysilicon layer as the structural layer and anchored metal probe pads. The analytical and numerical models were then validated experimentally by performing optical microscopy studies on the fabricated microgripper at atmospheric pressure. Each microgripper arm had a maximum in-plane deflection of around 2 μm at 3 V, resulting in a total microgripper opening of 9 μm. This is in line with the design requirement of 8 μm for securing and characterising a RBC. With an actuation voltage of up to 3 V, the size range of objects that can be manipulated with the fabricated microgripper is 5 to 9 μm. The analytical, numerical and experimental results for the deflection of the microgripper arm tips are all in good agreement, ensuring the reliability of the developed models to predict and optimise the performance of a polysilicon electrothermal microgripper at the design stage, prior to fabrication. This is considered an important contribution of this work since it is not always feasible to iterate on fabricated device designs (due to the costs and lead times involved), and thus establishing accurate analytical and computational models of the electrothermal actuator is considered essential to the design process.

Future work will focus on the enhancement of the presented microgripper design to include force feedback. This will be achieved by the integration of capacitive force sensors into the design to enable both contact detection as well as gripping force measurements. Contact detection is important to protect both the microgripper arms and the cell from damage. Cell grasping and releasing will be achieved by applying a sequence of actuation voltages, and the obtained gripping force feedback will help to define cell membrane parameters such as elasticity properties. The microgripper can serve as a diagnostic tool based on the expected differences in the obtained gripping force profiles for healthy and diseased cells. Such a tool can provide a quantitative investigation of the effects of disease states on the mechanical response of RBCs as well as valuable information about the disease status of patients. Moreover, due to the size and stiffness variations of the cells to be characterised, a single fixed grasping voltage could often cause either unsecured grasping or cell rupturing from excessively applied forces, thus necessitating reliable closed-loop force-controlled micrograsping to accommodate these variations.

## Figures and Tables

**Table 1:** Geometrical parameters of the fabricated microgripper.

Parameter	Value	Unit
Length of hot arm, $L_{h}$	200	μm
Length of cold arm, $L_{c}$	154	μm
Length of flexure, $L_{f}$	46	μm
Length of connector, $L_{g}$	3	μm
Length of gripping arm, $L_{a}$	203	μm
Width of hot arm, $w_{h}$	9	μm
Width of cold arm, $w_{c}$	55	μm
Width of flexure, $w_{f}$	9	μm
Width of gripping arm, $w_{a}$	9	μm
Thickness of silicon substrate	20	μm
Thickness of silicon nitride	0.6	μm
Thickness of air gap	2.75	μm
Thickness of dimples	2	μm
Thickness of Poly2	1.5	μm
Thickness of Metal	0.5	μm

**Table 2:** Material properties utilised in this work as obtained from the Coventorware^®^ Materials Library for the PolyMUMPs^TM^ fabrication process. All properties in this table are given at room temperature (300 K) and all references to polysilicon refer to the Poly2 layer.

Property	Value	Unit
Density of polysilicon	2.23	g/(cm)${}^{3}$
Young’s modulus of polysilicon, *E*	158	GPa
Poisson’s ratio of polysilicon, ν	0.22	-
Thermal expansion coefficient of polysilicon, α	2.80	μm/mK
Specific heat capacity of polysilicon, *c*	100	J/kgK
Thermal conductivity of polysilicon, $k_{p}$	32	W/mK
Thermal conductivity of air, $k_{a}$	0.0262	W/mK
Thermal conductivity of silicon nitride, $k_{n}$	25	W/mK
Electrical resistivity of polysilicon	30	μ· Ω· m
